# Occult spinal CSF leak: to patch or not to patch—a case-based update

**DOI:** 10.1007/s00381-025-07094-8

**Published:** 2025-12-22

**Authors:** Jiashu Wang, Amine P. Thomé, Allan L. Brook, Jocelyn C. Ronda, Andrew J. Kobets

**Affiliations:** 1 Albert Einstein College of Medicine, Bronx, NY USA; 2 Faculdade de Ciências Médicas de São José Dos Campos, São Paulo, Brazil; 3 Department of Radiology, Montefiore Medical Center, Bronx, NY USA; 4 Department of Pediatrics, Massachusetts General Hospital, Boston, MA USA; 5 Department of Neurosurgery, Montefiore Medical Center, Bronx, NY USA

**Keywords:** Cerebrospinal fluid leak, Epidural blood patch, Pediatric, Spontaneous intracranial hypotension, Case report

## Abstract

**Purpose:**

Spontaneous intracranial hypotension (SIH) is a rare condition in the pediatric population with a classic clinical presentation of severe orthostatic headaches that improve when the patient is lying down. The cause of SIH is cerebrospinal fluid (CSF) leakage either around the brain or spinal cord. When working up a patient with suspected SIH, an MRI is done, which aims to visualize the location of the leak. Visualization of the leak on imaging confirms the diagnosis of SIH and can guide localized treatments like epidural blood patch (EBP) or surgery. There has never been a reported case of a pediatric patient that presents with classic symptoms of SIH but shows completely normal findings on brain and spine imaging. Cases of adult occult SIH (oSIH) have been successfully treated with an empiric EBP; however, the use of an empiric EBP in a child with suspected oSIH has not been reported before.

**Methods:**

We describe the clinical features and management of a pediatric patient with an oSIH. A literature review was done for English articles, using PubMed, to identify previously reported cases of SIH and oSIH treated with empiric EBP.

**Results:**

A 9-year-old patient presented with worsening severe orthostatic headaches, nausea, phonophobia, a sensation of ear fullness, slight cognitive changes, and complete resolution of headaches when laying down. She received multiple MRIs of the brain and spine, which all found no abnormalities. She was treated for suspected post-viral syndrome and later for suspected migraines but had brief to no symptomatic relief. Her pain continued to worsen, debilitating her and causing her family distress. After being referred to our clinic, she was diagnosed with an oSIH and was treated with an empiric lumbar EBP. The patient made a profound recovery with a complete and sustained resolution of her symptoms. This shows the first pediatric case of a clinically suspected occult CSF leak with normal brain and spinal imaging, that was treated successfully with an empiric EBP after conservative treatments failed.

**Conclusion:**

While epidural blood patch has become a routine treatment option for SIH and a proven treatment in adult oSIH, its use in pediatric oSIH is still unclear due to the rarity of this disease. This case shows that an empiric EBP is a viable treatment option in pediatric oSIH. In situations where clinical suspicion for oSIH is high, empiric EBPs should be considered, since they have a relatively low procedural risk and can offer sustained and significant symptomatic improvement.

## Introduction

Spontaneous intracranial hypotension (SIH) is a rare condition characterized by severe orthostatic headaches that typically improve when the patient is lying down. This hallmark symptom helps distinguish SIH from other headache disorders. Diagnosis is often supported by MRI of the brain or spine demonstrating cerebrospinal fluid (CSF) leakage [1, 2]. When a leak is visualized, treatment may involve targeted interventions such as epidural blood patch (EBP) and surgery.

Past studies have shown that when adult patients present with classic signs of SIH, but imaging is unremarkable, they can be successfully treated with empiric EBP. However, due to the rarity of the disease in children, there are no cases reported of a pediatric patient with the clinical signs of SIH but explicitly normal brain and spine imaging.

In this report, we describe a pediatric patient with a textbook SIH presentation but without radiographic evidence of CSF leakage. We will explore the diagnostic challenges of diagnosing occult SIH (oSIH), the management of oSIH in adults, and the use of an empiric EBP in pediatric oSIH.

## Methods

A literature review of previously reported SIH and oSIH treated with empiric EBP was done in PubMed with the search terms, (“spontaneous intracranial hypotension” OR “spinal cerebrospinal fluid leak” OR “CSF leak”) AND (“pediatric” OR “children” OR “adolescent”) AND (“epidural blood patch” OR “blood patch” OR “EBP”). Papers included case reports, case series, and retrospective studies. From each included article, the following variables were extracted: patient demographics, clinical presentation, image findings, treatments, and treatment outcomes.

## Historical background

Spontaneous intracranial hypotension (SIH) was first recognized as a distinct clinical entity in the early twentieth century, when Schaltenbrand and later George described patients with orthostatic headaches caused by low cerebrospinal fluid (CSF) pressure. Over time, advances in imaging, particularly magnetic resonance imaging (MRI), have greatly improved the recognition of this syndrome by identifying typical radiographic findings such as diffuse pachymeningeal enhancement, venous distention, and subdural fluid collections [3].

The pathophysiology of SIH is most often attributed to spontaneous spinal CSF leaks that reduce intracranial CSF volume and pressure, leading to compensatory venous engorgement and brain sagging [4]. While the majority of cases occur in adults, pediatric presentations are rare, with only isolated case reports and small series available in the literature [5]. This rarity is likely multifactorial; children have more elastic meninges, lower baseline CSF pressure, and fewer degenerative or connective tissue changes predisposing to dural tears compared with adults. Moreover, pediatric patients often have difficulty describing the orthostatic nature of their symptoms, which can delay recognition and diagnosis.

In children, reported CSF leaks are most often associated with congenital abnormalities (such as meningeal diverticula or dural ectasia) or secondary causes including trauma, post-surgical changes, or lymphatic malformations leading to CSF-lymphatic fistulas [6]. These complex fistulas can cause spontaneous leakage of CSF into the lymphatic system, a mechanism increasingly recognized through advanced myelographic imaging. However, the small caliber of pediatric spinal anatomy, motion artifacts, and the need to limit radiation exposure make leak localization particularly challenging in this population.

Historically, SIH was underdiagnosed due to its nonspecific presentation and lack of clear precipitating events. Early diagnoses relied primarily on lumbar puncture and measurement of low CSF opening pressure, but these findings are now known to be absent in many patients. With the advent of modern MRI and myelographic techniques, such as digital subtraction myelography and dynamic CT myelography, clinicians can better identify sites of spinal CSF leakage [6].

The concept of occult SIH (oSIH) emerged when patients presented with classical orthostatic headaches but normal imaging findings. This phenomenon, initially described in adults, challenged the previous diagnostic criteria and led to the adoption of empiric therapeutic approaches such as the epidural blood patch even in the absence of radiographic confirmation [7]. However, pediatric oSIH remains exceptionally rare, and the role of empiric blood patching in this population has not been clearly defined.

## Clinical presentation

The classic presentation of SIH is diffuse, occipital, or frontal headaches that are almost always orthostatic. Rarely patients can present without headaches. Other common presenting symptoms are nausea and/or vomiting, neck pain and/or stiffness, hearing disturbances and other ear-related symptoms, and dizziness. More uncommonly, patients present with visual symptoms, movement disorders, and cognitive changes [4].

In many cases of SIH, there is an identifiable cause of CSF leakage, such as a history of lumbar puncture or congenital anomalies like Chiari malformations or lymphatic malformations. However, CSF leaks can also have an unknown cause. In 2017, Schievink proposed four types of CSF leaks associated with spontaneous intracranial hypotension (SIH): dural tears, meningeal diverticula, direct CSF-venous fistulas, and indeterminate/unknown sources [8]. This classification emphasizes the importance of a detailed medical history in patients with characteristic SIH symptoms [9].

## Diagnosis

SIH is diagnosed using criteria from the International Classification of Headache Disorders-3 (ICHD-3). It must be an orthostatic headache and must show at least one of the following: low CSF pressure (< 60 mm) and evidence of CSF leakage on imaging [10]. Based on recent studies, the absence of these criteria does not seem to have enough sensitivity to rule out SIH. Looking at the reported cases of SIH, low CSF pressure is seen in 34–93% of patients. In a meta-analysis done of 38 articles including 2078 patients, between 2 and 88 years old, CSF leakage on brain magnetic resonance imaging (MRI) was present in only 19% of patients. Evidence of CSF leak in the extradural space on computed tomography (CT) myelography, spinal MRI, radionuclide cisternography, magnetic resonance (MR) myelography, and digital subtraction myelography (DSM) was seen in 48–76% of cases [4]. When patients have the classic symptoms of SIH, no evidence on imaging, and no other medical cause, it is called occult SIH (oSIH).

## Management

The first line treatment for SIH is conservative management like hydration and bedrest. If those do not offer the patient symptomatic relief, a second option is a trial EBP, due to their potential for dramatic symptomatic improvement [11]. For cases of oSIH, empiric EBP is a viable treatment option. However, the use of empiric EBP in pediatric oSIH is less understood due to the lack of pediatric case reports of oSIH. This case represents the first pediatric patient presenting with spontaneous oSIH, with no abnormalities on brain or spinal imaging, who was treated with an empiric EBP.

In some pediatric cases of image-confirmed SIH, the placement of an epidural blood patch (EBP) has been shown to successfully manage symptoms. In a series of cases in children with a history of lymphatic malformations and intraspinal CSF-lymphatic fistulas, with CSF leakage confirmed on imaging, 2 of 4 patients initially received an EBP without symptomatic improvement and were later treated with surgery or liquid embolization. One of 4 patients had symptomatic remission from an EBP, and 1 of 4 patients was initially treated with surgery [12]. In another pediatric reported case of image-confirmed SIH, the 5-month-old patient had no history suggesting the cause of her CSF leak. She presented with headache, ataxia, and hearing loss. After receiving an EBP, her headache and ataxia completely and dramatically resolved, with no improvement in her hearing [5]. These cases show that EBP may have a high rate of failure but may offer total symptom relief to some patients, while being less invasive than other treatment options. There are few cases reported of pediatric SIH in the literature, with most being single cases or reviews of up to 6 other cases. Due to the rarity of this disease, a large-scale study has not been done so far (Table 1).

**Table 1:** Clinical, imaging, and management characteristics of previously reported cases of image-confirmed pediatric spontaneous intracranial hypotension (epidural blood patch (EBP))

Paper	Study type	Patient demographics	Clinical features	Imaging findings	Management	Outcomes
Bladen et al. 2007 [13]	Case report	M, 16 y	4 weeks of sudden onset and worsening occipital postural headache	MRI showed signs of intracranial hypotension	Autologous lumbar EBP	Complete symptom resolution at 6 months
Puget et al. 2007 [14]	Case report	F, 12 y with a history of Mafran syndrome, sacral dural ectasia, and tonsillar herniation	Postural headache	MR myelography showed CSF leak at S1	Headache was initially treated with posterior fossa decompression with worsening of symptoms. Then, after MR myelography, received EBP × 2 at S1	Resolution of symptoms after EBP × 2
Uysal et al. 2008 [5]	Case report	F, 5 y	1 month of daily positional headache, sudden onset hearing loss, ataxia	MRI showed signs of intracranial hypotension	5 ml EBP at L3-L4 × 2	Complete resolution of headache and ataxia with first blood patch, no improvement in hearing. Also, no improvement after second blood patch
Adler et al. 2011 [9]	Case report	F, 7 y with a history of Gorham-Stout disease and a posterior fossa decompression and duraplasty	Worsening postural headaches	MR showed signs of intracranial hypotension	Targeted transforaminal blood patch × 3. Liquid embolization of the receiving cavity	Significantly improved headaches a few days following treatment. At 6 months post-treatment, recurrence of mild positional headaches and evidence of intracranial hypotension on MR
Schievink et al. 2013 [15]	Case series (n = 24)	Mean age = 14.3 years (2–19) 18 girls, 6 boys	23 of 24 (96%) had positional headache, the other 1 of 24 (4%). had nonpositional headaches. 16 patients (67%) had nausea/emesis, 8 (33%) had aural symptoms, 8 (33%) had neck pain/stiffness, 8 (33%) had dizziness, 6 (25%) had visual symptoms. Other symptoms include photophobia/phonophobia, low back pain, confusion, hypersomnolence, and lumbrosacral radiculopathy	MRI showed signs of spontaneous intracranial hypotension in 19 patients (79%). Spinal imaging showed CSF leak in 22 patients (94%)	After bed rest and hydration, 23 (96%) patients were treated with EBP	One patient’s symptoms resolved with conservative management. 9 patients (39%) had permanent resolution of symptoms. 13 other patients (57%) had temporary improvement with recurrence of symptoms. 8 patients (35%) underwent fibrin glue injections directed at the leak. 11 patients (48%) underwent surgery. At follow up at varied times, 2 patients (9%) developed recurrent symptoms of SIH, both were treated successfully with surgery
Schönberger et al. 2017 [16]	Case report	M, 15 y	3 months of postural headache	MRI showed signs of intracranial hypotension	EBP at L1 bilaterally, L2 and L3 on the right side, L4 bilaterally. A total of 40 mL of autologous blood mixed with 6 mL of Solutrast 250 M was used	Symptom resolution after a few days of bed rest. Continued resolution of symptoms at 1 and 3 years
Önal et al. 2018 [17]	Case report	F, 13 y with a history of hypoglycemic convulsions and syncope attacks	Hypoglycemia, coma, convulsive seizures, dysarthria, syncope episodes independent of hypoglycemia, 2 h of severe biparietal headaches in the mornings	CT myelography showed two CSF leaks	Treatment for hypoglycemia, autologous EBP at L2	Complete control of her episodes of syncope, headache, and hypoglycemia. Hypoglycemia with dysarthria recurred after two months due to EBP failure. Neurological symptoms and hypoglycemia remained after EBP × 2. Treated with truncal vagotomy and partial pancreatectomy. Patient developed diabetes mellitus post-operatively controlled with insulin. Resolution of symptoms at 4 years
Yokoi et al. 2020 [18]	Case report	F, 14 y with a history of Gorham-Stout disease	1 year of spontaneous mechanical thoracic back pain, 2–3 months of positional headaches associated with Valsalva maneuvers	CT showed signs of intracranial hypotension	Direct surgical repair and fixation of T6-T11	Persistent positional headaches, at 6 months CT showed improvement of cerebellar sagging
Ghosh et al. 2021 [19]	Exemplary case	F, 12 y with history of migraines	1 month of increasing, throbbing bifrontal headache, bettering with laying supine	MRI showed signs of intracranial hypotension	EBP at L1-L4 × 2	Patient continued to have orthostatic headaches and episodes of syncope, given fludrocortisone with improvement of symptoms
	Case series (n = 4)	Mean age = 15 (11–17) 1 boy, 3 girls 3 had a history of migraines	Throbbing headache, severe pulsating pain in the morning	For 3 of 4, CT myelography showed dural leak. For the other 1 of 4, MRI showed signs of intracranial hypotension	Unknown	Unknown
Soderlund et al. 2021 [20]	Case report	F, 9 y with a history of kaposiform lyphangiomatosis	Several years of chronic headaches, new horizontal diplopia due to an abducens nerve palsy, and new evidence that headaches improved when lying supine	MRI showed signs of intracranial hypotension	Targeted transforaminal 4 cc EBP, later surgical ligation	After initial blood patch she still had headaches. They completely resolved after surgery. At 15 months post-treatment, complete resolution of headaches
Fric et al. 2024 [12]	Case report	F, 9 y with a known history of a lymphatic malformation	Postural headaches, increasing with upright position, nausea, poor cooperation, lethargy, blurred vision, unsteadiness, dizziness	MRI showed signs of intracranial hypotension	Image-guided percutaneous autologous 5 ml blood patch into the foramen of C7/T1	Resolution of cerebellar sagging on MRI. At 7- and 15-months post-procedure, no neurologic symptoms except slight fatigue and polyarticular pain, and pain in legs after excessive motion
Fang et al. 2025 [21]	Case report	F, 14 y with history of Lennox-Fastaut syndrome, intractable epilepsy, spastic quadriplegia, left frontal ventriculoperitoneal shunt, developmental and intellectual delay	Sudden onset right eye ptosis and gaze deviation, and moving more “unsteady”	MRI of the brain showed evidence of intracranial hypotension and increased protein in CSF	Bed rest, hydration, and resumption of home medication therapy	Complete and spontaneous resolution of symptoms at 3 months
Gavin et al. 2025 [22]	Case report	M, 14 y with history of a fall onto his coccyx 3 days prior	Positional headache only when sitting or standing upright, nausea, vomiting	MRI of the spine showed large subarachnoid cyst and CSF accumulation	Bedrest, supine positioning, caffeine	Discharged after 2–3 weeks of conservative treatment, headaches resolved 2 weeks after discharge

The use of empiric EBP for adult patients who have orthostatic headaches but neither a low CSF pressure nor evidence of CSF leak on imaging has also been proven somewhat successful (Table 2). A 2011 study by Cho et al., showed that 25 of 56 (44.6%) adult patients had symptoms of SIH but showed no CSF leakage on imaging. These 25 patients were treated with an empiric EBP, with 19 patients receiving EBP via a lumbar epidural route (L3-L4) and 6 patients receiving EBP via an upper thoracic epidural route (T4-6). 12 of 19 (63.2%) patients that received a lumbar empiric EBP required more than 2 additional EBPs, while only 6 patients had no recurrence of residual symptoms after the first EBP. All 6 patients who received an upper thoracic empiric EBP had resolution of symptoms [23]. More recently, in a 2023 study, patients who were between 16 and 64 years old, Choi et al. found that 40% of their patients with clinically suspected SIH showed no abnormalities on brain and spine imaging. In these oSIH patients, they treated with empiric EBPs and found that 66.7% reported an improvement in symptoms by discharge, 90.5% reported an improvement in symptoms at 3 months, and 52.4% had complete remission at 3 months [7]. This shows that oSIH is not uncommon in the adult population, but the efficacy of empiric EBPs is not entirely understood and varies patient-to-patient. However, due to the relatively low risk of this procedure, a therapeutic empiric EBP is still beneficial to try in patients with no CSF leakage on imaging, before pursuing a more invasive treatment 2.

**Table 2:** Clinical, imaging, and management characteristics of previously reported cases of adult occult spontaneous intracranial hypotension (epidural blood patch (EBP))

Paper	Study Type	Patient demographics	Clinical features	Imaging findings	Management	Outcomes
Cho et al. 2011 [23]	Case series (*n* = 56)	Mean age = 39.6 y (22–69) 23 men, 33 women	55 (98.2%) patients demonstrated orthostatic headache	MRI showed diffuse pachymeningeal enhancement in 48 patients. CT myelography located CSF leak site in 37 patients (66%)	5 patients were treated with bed rest and IV saline. 31 patients received targeted EBP. 19 patients received blind EBP via lumbar epidural route (L3-L4). 6 patients received blind EBP via upper thoracic epidural space (T4-6)	27 of the 31 patients (87%) that received targeted EBP had symptom relief with no recurrence. 12 of the 19 patients (63%) who received a blind EBP via lumbar route had persistent headaches and required more than 2 additional EBPs. 6 patients who received blind EBP via thoracic route had resolution of symptoms
Choi et al. 2023 [7]	Case series (*n* = 191)	Mean age = 40 y (33–52)	Orthostatic headache	121 patients received imaging. 47 (38.8%) patients had normal brain MRI, and CT or MR myelography	21 of the 47 patients with normal imaging were treatment-naïve. These patients received empiric EBP	17 patients received one EBP with 76.5% reporting symptom resolution. 2 patients required a second EBP with remission of symptoms. 2 other patients required a third EBP with symptom resolution in one patient

## Prognosis and outcomes

Like many other patients, the patient presented here was initially misdiagnosed and therefore did not receive symptomatic relief for multiple months. This experience is not uncommon, as many patients with spontaneous SIH are initially misdiagnosed. Commonly, they are first diagnosed with migraines, meningitis, and psychogenic disorder [11]. If imaging is able to confirm a CSF leak, the correct diagnosis of SIH is easier to reach; however, with an occult SIH, it can be even more challenging to reach the right diagnosis.

Similar to other cases of clinically suspected oSIH, in the case presented, the clinical team chose to proceed with an empiric EBP based on the patient’s textbook SIH presentation, debilitating symptoms, and failure to respond to prior medical management, despite the absence of radiographic abnormalities. Importantly, the procedure was carried out by a highly skilled interventional radiologist with extensive experience in this technique. In less experienced hands, however, empiric EBPs can be hazardous. The most significant risk is intrathecal (intradural) injection of blood, which can lead to inflammation, nerve root injury, and even the formation of intraspinal cysts. This procedural risk contributes to clinician hesitation, especially in the absence of radiographic confirmation of a CSF leak.

Our case supports that empiric lumbar epidural blood patch (EBP) is a successful treatment in managing pediatric oSIH. In this case, the patient’s dramatic and sustained response to empiric treatment despite the complete absence of radiologic evidence of a CSF leak shows that this is an important treatment to consider pursuing in suspected oSIH. This highlights the critical importance of clinical judgment, particularly when the patient’s symptoms are disabling, classic in presentation, and refractory to medical therapy. In such situations, a trial of empiric EBP may be warranted even in the absence of imaging confirmation.

One factor that may have contributed to the success of this case was the use of a high-volume patch (> 15 mL). Larger volumes can coat a broader area of the dura and are more likely to tamponade a leak when the exact site is unknown. This approach aligns with prior reports suggesting that the volume and extent of spread can be just as important as the location when treating presumed CSF leaks empirically [3, 24]. In some cases, the volume of EBP is can vary because it can be adjusted based on the patient’s feedback. When patients report pain or discomfort in the lower back or radiating to the buttocks or legs, the injection is temporarily stopped and resumes when the symptoms subside [25]. This can cause slight variation in the patch volume in certain cases.

## Exemplary case description

A 9-year-old female with a history of a pituitary cyst and a mild cervical spine (C-spine) injury sustained in a motor vehicle accident 2 years prior, presented with approximately 6 weeks of progressively worsening, bilateral frontal, stabbing headaches. The headaches were positional in nature, markedly improving when lying down, and reached a severity of 10/10 at the time of presentation. She had several episodes of vomiting, phonophobia, a sensation of ear fullness, and subtle cognitive changes (e.g., reported new difficulty with reading). She had no headache symptoms since her C-spine injury, until this presentation, so there was low clinical suspicion that the cause of her headaches was from her previous injury. Her symptoms were unresponsive to over-the-counter medications such as acetaminophen and ibuprofen. The frequency and the intensity of the headaches progressed over time and significantly impaired her daily functioning. She became bedbound, missed weeks of school, and was frequently crying herself to sleep due to the severity of the pain. Her family grew increasingly distressed by the ineffectiveness of prior treatment efforts.

**Fig. 1:**
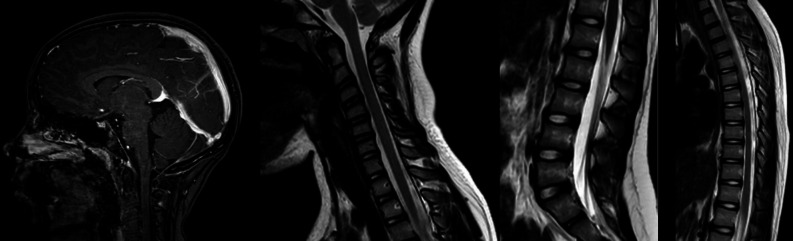
a-d Pre-procedure imaging: Brain MRI (**a**) and sagittal T2-weighted MRI of the cervical (**b**), lumbar (**c**), and thoracic (**d**) spine with no signs suggesting intracranial hypotension

The first episode of headaches was 2.5 months prior to presentation at the hospital. She developed malaise and nasal congestion and was prescribed treatment for sinusitis with doxycycline. She subsequently experienced an episode of severe headache. She described the pain as sharp and associated with dizziness and a “floppy” sensation in her legs. She was diagnosed with a post-viral syndrome and her symptoms resolved with days of bedrest. However, she then experienced several additional episodes of headaches, dizziness, and “floppy” legs, each improving with bedrest. After these episodes, she was diagnosed with migraines and was prescribed a prednisone taper and rizatriptan. The prednisone and rizatriptan did not improve her symptoms. She was additionally treated with intravenous medications including ketorolac, magnesium, prochlorperazine, metoclopramide, and sodium chloride which also did not provide any relief of her symptoms. She underwent a brain MRI with and without contrast and noncontrasted MRIs of her cervical, thoracic, and lumbar spine. The imaging was unremarkable and showed no spinal epidural collection (Fig. 1).

In the month prior to presenting in our clinic, the patient was experiencing debilitating daily headaches, whenever she was sitting or standing. The pain increased in severity and had become harder to relieve by lying down. Due to the severe pain, the patient had difficulty eating, drinking, sleeping, sitting, standing, walking, and attending school. This caused her to miss weeks of school. The patient’s mother consulted with multiple specialists around the country, and they raised concerns for a spontaneous CSF leak. Not being able to find timely care locally, she reached out to our team given our experience managing pediatric patients at the Children’s Hospital at Montefiore.

At our clinic, the patient was evaluated for a CSF leak and admitted the same day. An MRI brain with and without contrast and an MRI of the cervical, lumbar, and thoracic spine without contrast were all unremarkable and showed no findings to suggest intracranial hypotension (Fig. 1). The patient did not receive a dynamic CT myelogram as her team thought it would be too low yield, because there was no evidence for CSF venous fistulas on MRI, and would therefore her to unnecessary radiation. However, due to her classic presentation of orthostatic headaches, the severity of her clinical experience, the lack of treatment response to prior medical therapy, and our team’s experience and comfort managing these patients, there was high clinical suspicion that she had SIH, and we embarked upon treatment the following day. She underwent a CT guided empiric, high-volume (~ 15 CC) epidural blood patch under monitored anesthesia care with no complications.

The patient had a profound response to treatment with resolution of her headaches within days of treatment. She was able to resume normal daily activities like eating, drinking, sitting, standing and walking without pain. She returned to school and was able to read again without any difficulty. She is presently more than 4 months from her EBP and has no headaches or residual neurological symptoms.

## Conclusions

There is sufficient evidence supporting the use of EBP in pediatric SIH, and the use of a trial empiric EBP in adult oSIH. This case shows that empiric EBP may be a valid and safe treatment option for pediatric patients with suspected oSIH, who do not improve with conservative treatment like bed rest and oral hydration. While each patient must be carefully evaluated on an individual basis, the relatively low procedural risk, when performed by an experienced clinician, is often outweighed by the potential for dramatic and sustained symptomatic improvement.

New advanced myelographic techniques, such as digital subtraction myelography (DSM), dynamic CT myelography (CTM), and decubitus CTM without a dynamic component, may be a part of the future of imaging otherwise occult CSF leaks. These techniques are able to obtain images very soon after contrast injection, improving their ability to visualize leaks. Individual case reports that use these imaging techniques to localize leaks in children have shown positive results but are rare due to the rarity of SIH in children. Madhavan et al. in 2024, reviewed 12 pediatric patients with SIH whose CSF leaks were successfully localized using myelographic imaging techniques. In this review, different etiologies of CSF leak, such as lateral dural tears, CSF fistulas, and ventral dural tears, were visualized on advanced myelography. All the patients underwent successful treatment with 11 of the 12 patients reporting near-complete or complete symptom resolution [6]. These new techniques could potentially help us visualize otherwise occult leaks, but the radiation exposure to the patients in these techniques is not insignificant, especially to pediatric patients. In suspected occult SIH cases, an empiric, large volume patch may be less risky than the radiation exposure while achieving the same ultimate result. If an empiric EBP causes symptom resolution, there would be no need to confirm the exact leakage point prior to treatment. While the risks of benefits of each case must be considered individually, these new myelographic imaging techniques may be most useful after a patient does not show improvement after receiving an empiric EBP.

Ultimately, this case and associated literature review shows that EBPs are an effective treatment in pediatric SIH, empiric EBPs are effective in adult oSIH, and empiric EBPs should be pursued in pediatric patients with a classic presentation of SIH but otherwise non-diagnostic imaging. When performed by an experienced clinician, empiric lumbar EBP can offer safe, meaningful, and sustained symptom relief, even in the absence of radiographic confirmation. Further studies are needed to explore novel diagnostic options for these patients and less invasive treatment strategies. However, in our experience, empiric epidural blood patches are a feasible treatment option for pediatric occult CSF leaks, for which the risks and benefits must be weighed on a case-by-case basis.

## Data Availability

No datasets were generated or analysed during the current study.
